# MelTrans: Mel-Spectrogram Relationship-Learning for Speech Emotion Recognition via Transformers

**DOI:** 10.3390/s24175506

**Published:** 2024-08-25

**Authors:** Hui Li, Jiawen Li, Hai Liu, Tingting Liu, Qiang Chen, Xinge You

**Affiliations:** 1 School of Electronic Information and Communications, Huazhong University of Science and Technology, Wuhan 430074, China; d201980610@hust.edu.cn (H.L.); xingeyou1975@hust.edu.cn (X.Y.); 2 National Engineering Research Center for E-Learning, Central China Normal University, Wuhan 430079, China; jiawenli@ccnu.edu.cn (J.L.); hailiu0204@ccnu.edu.cn (H.L.); 2022214724chenqiang@mails.ccnu.edu.cn (Q.C.)

**Keywords:** speech emotion recognition, feature extraction, Transformer, deep learning

## Abstract

Speech emotion recognition (SER) is not only a ubiquitous aspect of everyday communication, but also a central focus in the field of human–computer interaction. However, SER faces several challenges, including difficulties in detecting subtle emotional nuances and the complicated task of recognizing speech emotions in noisy environments. To effectively address these challenges, we introduce a Transformer-based model called MelTrans, which is designed to distill critical clues from speech data by learning core features and long-range dependencies. At the heart of our approach is a dual-stream framework. Using the Transformer architecture as its foundation, MelTrans deciphers broad dependencies within speech mel-spectrograms, facilitating a nuanced understanding of emotional cues embedded in speech signals. Comprehensive experimental evaluations on the EmoDB (92.52%) and IEMOCAP (76.54%) datasets demonstrate the effectiveness of MelTrans. These results highlight MelTrans’s ability to capture critical cues and long-range dependencies in speech data, setting a new benchmark within the context of these specific datasets. These results highlight the effectiveness of the proposed model in addressing the complex challenges posed by SER tasks.

## 1. Introduction

Speech emotion recognition (SER) is a fundamental problem in the field of human–computer interaction (HCI). It aims to give high-accuracy speech emotion classification predictions for emotion researchers. Given that the emotional states of everyday human life play an important role in interpersonal communication, increasing attention is being attached to the study of speech emotion. As an upstream task, SER has a wide range of applications and has garnered significant attention in the field of human–computer interaction (HCI) [1,2,3,4,5,6,7,8], self-driving vehicles [9], intelligent voice assistants and e-learning [10]. Excellent performance on SER can help downstream tasks. Automatic emotion classification technology can significantly enhance the safety and comfort of autonomous driving [11]. By monitoring the driver’s emotional state, the system can detect signs of fatigue, stress or aggression, and respond appropriately to ensure safe driving conditions. Additionally, the practical application of speech emotion recognition (SER) should consider ethical issues related to users [12]. However, SER has long been a challenging task because of the inherent ambiguous nature of emotions and background noise.

Over the past decades, numerous works on SER have been performed. Among them, deep learning (DL) methods have emerged as a rapidly advancing field that has demonstrated remarkable effectiveness and promising results [13]. Therefore, we only mention DL-based speech emotion classification methods. They can be roughly categorized into two families: relevance of information (RoI)-based methods and speech-only usage (SOU) methods.

For RoI-based methods, the LSTM-GIN model [14] is mostly adopted because it defines connections in accordance with temporal relations between speech frames. Liu et al. [15] introduced an innovative SER framework that employs an adversarial joint loss strategy, combined with a cascaded attention network, to effectively extract meaningful emotional features. The long-distance dependencies of temporal features in targeted regions are captured.

Compared with the RoI-based methods above, SOU methods are more advanced and promising because global-level signal information is utilized and sentiment analysis failures caused by feature selection bias are prevented. The most prevalent backbone of SOU methods is a convolutional neural network (CNN), such as ResNet [16], AlexNet [17] and SENet [18]. In [19], Lei et al. proposed an effective approach named MsEmoTTS, which taps into the relationship of emotional characteristics between different levels to achieve multiscale emotional speech synthesis. Speech and text-based speech synthesis methods were found to outperform audio- and text-based sentiment analysis methods, respectively, through extensive experiments. Makiuchi et al. [20] developed a temporal alignment mean–max pooling mechanism, a straightforward yet effective network architecture that enables fine-grained discourse capture, facilitating emotion calculation through textual information for cross-excitement scenarios. On the basis of the study of multimodal emotions in speech, Zhou et al. [21] devised an adaptive strategy to dynamically calculate the fusion weight of two modalities, leveraging local and global features for sentiment classification tasks. To address the inherent challenge of feature representation in language-based emotion recognition, Chen et al. [22] introduced a novel approach termed attention mechanism-based multiscale SER network (AMSNet). This pioneering framework integrates a parallel network architecture designed to effectively combine fine-grained frame-level features with coarse-grained utterance-level depth features. Furthermore, Feng et al. [23] presented a few-shot learning method for emotion recognition in spontaneous speech, utilizing a Siamese neural network with adaptive sample pair formation. The SER task exploits the excellent performance of the Transformer [24] encoder based on multi-head attention. Although SOU methods dramatically reduce the labor cost of dataset annotation and perform better than traditional methods, numerous challenges remain for SER.

### 1.1. Challenges

Given the inherent characteristics of voice signals, some challenges hinder the improvement in the accuracy of SER, which can be summarized in the following aspects.

1. Emotional subtleties: Detecting subtle emotional nuances in speech presents a significant challenge. Emotions are often expressed through minor variations in vocal attributes such as tone, pitch and intensity. These subtle cues can be easily overshadowed by more overt emotional expressions or obscured by background noise and other distortions. Consequently, accurately identifying and distinguishing these fine-grained emotional differences requires advanced analytical methods. Overcoming this challenge is essential for enhancing the sensitivity and accuracy of emotion recognition systems in capturing and interpreting subtle emotional signals.
2. Noisy background: Communications often have complex and various environmental backgrounds. Such environments often feature multiple overlapping sounds that complicate the task of isolating the target voice from the background noise. This interference not only diminishes the clarity of the speech signal but also obstructs the accurate identification of the speaker’s emotional state. Addressing this challenge is crucial for improving the effectiveness of SER systems in noisy conditions.

### 1.2. Observation and Insights

In the field of speech recognition, several spectrogram transformations can be applied to analyze single voices or crowds, including mel-, log- and bark-spectrograms [25]. Currently, the most widely used transformation method is the mel-spectrogram, which is designed to mimic the human ear’s perception of sound, providing a more natural representation of audio signals. This transformation emphasizes frequencies that are more critical to human hearing, thereby enhancing the performance of speech recognition systems [26]. Therefore, we utilize the mel-spectrogram in our approach.

**Figure 1:**
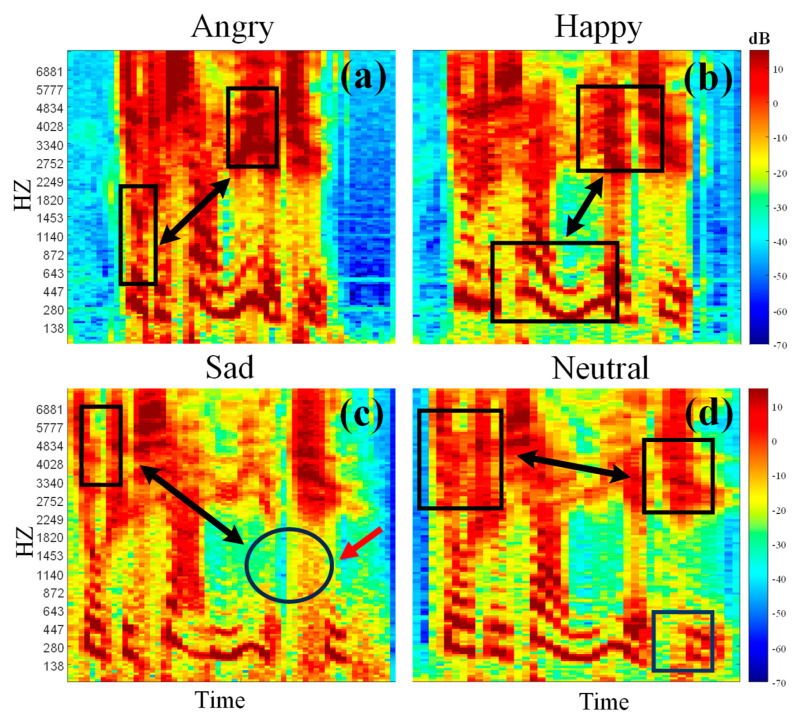
Mel-spectrograms of different speech emotions. Differences in the energy (dB) are reflected in the shade of color. Subfigures (**a**–**d**) represent different emotions: (**a**) angry, (**b**) happy, (**c**) sad, and (**d**) neutral. The black arrows indicate the presence of long-range dependencies in the speech signal, while rectangles highlight regions with high mel-spectrogram values, representing crucial cues. Circles denote regions with lower mel-spectrogram values, and the red arrows specifically point to these low-energy regions.

We find several characteristics that tackle the SER challenges by carefully observing the spectrogram of the voice signal. As shown in Figure 1, We selected four common emotions (happy, sad, angry and neutral) from two datasets to create mel-spectrograms. Each spectrogram is processed through segmentation, and the features vary from one to the other. Some emotions have a similar pitch, tone length, tone repetition and timbre. Their spectrograms appear similar but express different emotions. Nevertheless, some crucial features that can be leveraged for SER are always available.

**Key insight I:** Crucial cues of emotions. Emotions are not quickly categorized by surfaces. Some intra-class core features are distinguished from others. For instance, Figure 1a displays the mel-spectrogram of an angry speech emotion. Although the mel-spectrogram has multiple characteristics to look out for, while analyzing these features, they may introduce confusing information, which is a hazard for SER. This risk can be alleviated by recognizing the long-range dependencies of spectrum parts that are defined as significant characteristics.

**Key insight II:** Minuscule discrepancies in different emotions. For different mel-spectrograms of emotions, minor differences can be observed in most cases. For this special characteristic in SER, the perception of determinative fine-grained features is crucial. As shown in Figure 1, under the same statement, the mel-spectrogram of the anger emotion shows stronger energy than the others. The arrow pointing to the circle in the mel-spectrogram that represents sadness is weak. These inconspicuous but significant features can be easily neglected. Hence, some features are more meaningful and discriminative than other features.

The above findings can be summarized as the problem of finding the crucial cues and long-range dependencies. Specifically, we argue that recognizing the crucial cues of the same emotion, which often remain consistent across different settings, as well as the inconspicuous but significant long-range dependencies that are usually neglected by existing approaches, is essential. Thus, how to effectively utilize these two findings plays a significant role in improving the accuracy of SER. The motivation of our work is to design a SER model in which a small amount of key information in speech can be mined. To this end, we build a dual-stream model with a crucial cue stream for learning key features and a relationship stream for revealing long-range information. These two streams work together to facilitate the performance of SER.

### 1.3. Contributions

Unlike previous works on SER that focus on exploiting general emotion features, our work takes an insightful view to reveal the significant characteristics of speech. Our motivation consists of two parts: how to find the crucial cues of specific emotions and how to discriminate their variations to classify similar emotions. To leverage the findings that we observe, we propose a token-mask strategy that masks linguistic information and then predicts the masked areas, thereby improving the feature-learning capability of our model. 

The main contributions of this work can be summarized as follows:

1. An efficient MelTrans model is developed to leverage the findings we observe in speech emotion signals. To the best of our knowledge, the critical cues and long-range semantic relationships in voice signals are revealed for the first time. Then, a Transformer is leveraged to exploit the relationships among mel-spectrograms.
2. A dual-stream model is proposed to exploit crucial cues and long-distance relationship. Specifically, the crucial cue stream extracts the core representations in speech signals, while the relationship stream generates the long-distance relationship information of speech. Together, the two streams can make full use of the information in voice signals to form a core cue-aware neural network.
3. Experiments are conducted on IEMOCAP [27] and EmoDB [28] datasets. The results show that, on the same dataset, the proposed MelTrans model yields better performance than several methods, such as ICAnet and AMSNet, validating its effectiveness.

The rest of this paper is structured in the following manner. Current works related to SER are presented in Section 2. The details of our model are elaborated in Section 3. Section 4 provides the experimental results and discussion on different datasets. Section 5 discusses limitations and future work. Finally, we conclude our work in Section 6.

## 2. Related Work

### 2.1. Speech Emotion Recognition

Speech emotion recognition (SER) aims to analyze the emotional states of a person by their voice, which is beneficial for understanding human true emotions. In recent years, many SER methods [29,30] have been proposed. Zhu et al. [31] introduced a multilingual emotional text-to-speech (METTS) model, which addresses the inherent challenges associated with emotional transfer in text-to-speech systems, particularly concerning cross-speaker and cross-lingual scenarios. The METTS model brought a significant advancement in the field of emotional synthesis. Dong et al. [32] proposed a novel temporal relation inference network (TRIN) based on the attention mechanism. Zheng et al. [33] proposed a multiscale residual aggregation network to capture long-term variations in speaker characteristics to obtain significant information. Although these methods have achieved impressive performances for SER, they still have several limitations. For example, the comprehensiveness of feature extraction is always inadequate. To address this problem, Zou et al. [34] designed a co-attention module based on multilevel acoustic information to utilize in-depth audio information. However, this method neglected the balance between different granularities and the component relationship in the signal. Hence, Chen et al. [35] proposed SpeechFormer++ based on the Transformer framework. Although these existing studies have made consistent progress in SER, few works consider learning long-range dependencies in speech. Consequently, how to exploit long-range dependencies more effectively to improve the performance of SER is the main focus of our work.

### 2.2. Attention Mechanism

The attention mechanism [36] was first designed in the computer vision (CV) domain and then obtained good results first in machine translation tasks in the natural language processing (NLP) domain. Zhou et al. [21] proposed a multimodal fusion attention network for audio–visual emotion recognition. In [37], Kwon, S. proposed a conversational Transformer network, which learns context- and speaker-sensitive dependencies. Thanh et al. [38] introduced a pioneering contribution in the form of their pitch-fusion model, specifically tailored to address the nuances of tonal languages for SER. This innovative model harnesses the power of efficient cross-attention and self-attention mechanisms to effectively align pitch features with contextual acoustic features extracted from a state-of-the-art speech representation model, such as Wav2Vec 2.0. In this work, we observe that a few features of the different emotional components are strongly correlated in the mel-spectrogram, and these few features play a meaningful role in the analysis of speech emotions. The attention mechanism thus comes to our minds.

### 2.3. Transformer-Based SER

Originating from its success in CV and NLP, the Transformer architecture has been introduced into SER to learn representations in mel-spectrograms. Ref. [39] proposed a feature-fusion model (Dual-TBNet) that consists of two 1D convolutional layers, two Transformer modules and two Bidirectional Long Short-Term Memory (BiLSTM) modules. This structure can protect the speech information and realize strong robustness for the features. Ref. [40] fused spatiotemporal feature representations using convolutional neural networks (CNNs) and Transformer encoders to characterize SER, which effectively addressed the problem of efficiently extracting emotional features from speech dialogues. In this study, we use the Transformer architecture because it can capture the correlations between features.

### 2.4. Summary

Here, we observe that most existing techniques overlook key cues and subtle differences. In contrast, our approach leverages the Transformer architecture to successfully address these issues, providing a more nuanced analysis.

## 3. Proposed Method

**Figure 2:**
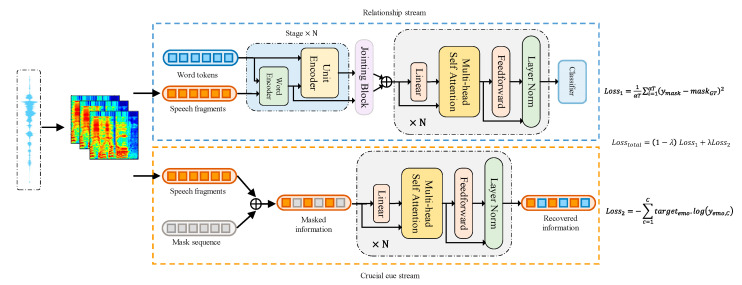
Dual-stream architecture of MelTrans. The crucial cue stream learns the crucial cues, and the relationship stream exploits the long-range-dependent relationships in speech signals.

We propose a dual-stream network dubbed SER via mel-spectrograms based on a Transformer (MelTrans). The framework of MelTrans is shown in Figure 2. Each stream in MelTrans solves a different subtask, and the streams are then fused to solve the final SER task. Specifically, the relationship stream takes the log-mel features as input to learn long-range dependencies in log-mel signals. The crucial cue stream takes the partially masked log-mel features as an input to predict the missing values. To learn the different scale features of the original signal, the relationship stream adds a word-encoder model. All the components of MelTrans are divisible. Therefore, our proposed components can be easily transplanted to the design of networks for other tasks. 

### 3.1. Crucial Cue Stream

The crucial parts of a speech signal not only simply contain a set of semantics and tones but also imply the whole statement’s contextual information and the relationships between semantics and tones. The word–level relationship is useful to identify and locate the connections between words. However, if this relationship becomes untrustworthy or nonexistent, then capturing a few key features is an important way to identify the sentiment of the statement. Therefore, our proposed MelTrans is designed to capture high-level relationships to improve the accuracy of SER. We propose a crucial cue stream, which is composed of Transformer blocks to analyze the crucial cues, so we mainly study how to improve the accuracy of recovering the masked values.

In the crucial cue stream, an original input speech fragment is represented as $c=\left[c_{1},c_{2},\ldots ,c_{T}\right]$, where T denotes the sequence length. Then, a binary mask sequence, which has the same dimension as the input sequence, is generated to perform the mask recovery task. A 1 in the mask sequence indicates that the corresponding time step needs to be masked, and a 0 indicates that it is retained; thus, the mask sequence can be expressed as $u=\left[u_{1},u_{2},\ldots ,u_{T}\right]$. 

For yielding input information that is partially masked before being fed into the model, the generated mask sequence should be merged with the original feature sequence.

The masked information $c_{m}$ is obtained simply by mask processing, which can be expressed as

(1) $$c_{m}=c⨀\left(1-u\right),$$

where ⨀ represents element-by-element multiplication. After $c_{m}$ is obtained, within the masking information embedding layer, the binary mask is projected to the dimensionality matching that of the original feature and is subsequently incorporated into the input that can be expressed as

(2) $$c^{\prime }=c_{m}+Linear\left(u\right),$$

where $Linear\left(•\right)$ is a linear layer that embeds occlusion information u into the input. Then, by employing the multi-head self-attention (MHSA) operation, the model can dynamically exploit the relationship between the known and masked parts. This operation is expressed as

(3) $$\bar{c}=MHSA\left(c^{\prime },c^{\prime },c^{\prime },u\right),$$

(4) $$c_{out}=LayerNorm\left(\bar{c}+c^{\prime }\right).$$

The acquired $c_{out}$ is fed into a feed-forward network (FFN) to capture higher-level features, thereby enhancing the comprehension of critical features, which are expressed as

(5) $$c_{out}^{\prime }=LayerNorm\left(FFN\left(c_{out}\right)+c_{out}\right).$$

### 3.2. Relationship Stream

*(1) Word encoder*

In this section, a network module based on a word-encoder and object-encoder structure is proposed to thoroughly learn the coarse- and fine-grained characteristics from speech signals. The structure will be introduced in detail. 

To capture the coarse-grained information in the original speech signal, we propose a word encoder to learn the feature. We first create several learnable word tokens: $c_{1}\in R^{N_{x}\times W_{1}}$ for stage 1, where $N_{x}$ indicates the approximate number of words in the statement. $c_{2}\in R^{N_{x}\times W_{2}}$ for stage 2 and $c_{3}\in R^{N_{x}\times W_{3}}$ for stage 3 are produced by the jointing block. Then, the input variable $s_{i}$ is segmented into $N_{x}$ non-overlapping intervals with uniform distribution.

(6) $$\left[m_{i1},m_{i2},\ldots ,m_{iN_{x}}\right]=NonOverlapping\left(s_{i},\frac{N_{i}}{N_{x}}\right),$$

where $NonOverlapping\left(•\right)$ represents the non-overlapping segmentation. $m_{ij}$ is the j-th non-overlapping segment of $s_{1}$ and $j\in \left[1,N_{x}\right]$. $c_{i}^{j}$ denotes the j-th word token in $c_{i}$, and $c_{i}^{j}$ is the updated value of $c_{i}^{j}$.

Ultimately, each word token learns coarse-grained characteristics of diverse segments. This operation is expressed as

(7) $$m_{ij}=s_{i}\left[\frac{N_{i}}{N_{x}}\times \left(j-1\right):\frac{N_{i}}{N_{x}}\times j\right]$$

$c_{i}^{j}$ is forwarded through the object encoder across various stages to harness coarse-grained information during the modeling process.

(8) $$c_{i}^{j}=MHSA\left(c_{i}^{j},m_{ij},m_{ij}\right),$$

where $MHSA\left(•\right)\;$ represents multi-head self-attention.

*(2) Object encoder*

For the object encoder, an original input speech signal is transformed into acoustic representations $s_{1}\in R^{N_{1}\times W_{1}}$, where $N_{1}$ is the number of frames and $W_{1}$ is the dimension of each frame embedded. To learn the information about consecutive frames in stage 1, we utilize an object encoder with a window $N_{o1}$ to extract the frame-grained features in $s_{1}$. This operation is expressed as

(9) $$\left[s_{i_{1}},s_{i_{2}},\ldots ,s_{i_{N_{i}}}\right]=Overlapping\left(s_{i},N_{oi}\right),$$

(10) $$s_{ij}=s_{i}\left[j-\frac{N_{oi}}{2}:j+\frac{N_{oi}}{2}\right],\;\;\;\;j\in \left[1,N_{i}\right],$$

where $Overlapping\left(•\right)$ represents the overlapping segmentation, the subscript “i” denotes stages 1–3 and $s_{i}\left[x:y\right]$ consists of the x-th to the y-th tokens of $s_{i}$. Subsequently, to enable the object encoder to consider coarse-grained information, we pass the learnable $c_{i}$ to each word encoder in each stage. Thus, the attention in each segment can be written as

(11) $$h_{i}^{jz}=Concat\left(c_{i}^{\left(z\right)},s_{ij}\right),$$

(12) $$g_{i}^{jz}=MHSA\left(x_{i}^{\left(j\right)},h_{i}^{jz},h_{i}^{jz}\right),$$

(13) $$s_{i}^{\left(j\right)}=Norm\left(g_{i}^{jz}+s_{i}^{\left(j\right)}\right),$$

where $h_{i}^{jz}\in R^{\left(1+N_{oi}\right)\times W_{1}}$ is the enhanced segment, $z=ceil\left[\frac{j\times N_{a}}{N_{i}}\right]$ rounds a number upward to its nearest integer and $j\in \left[1,N_{i}\right]$. The resultant x is then fed into an FFN that can be expressed as

(14) $$s_{i}=Norm\left(FFN\left(s_{i}\right)+s_{i}\right).$$

In the process from stages 1 to 3, each stage is dedicated to a different granularity, progressively transitioning from frames to consonants to words. The input features are denoted as $s_{i}\in R^{N_{i}\times W_{i}}$, and $\;i\in \left[1,3\right].$ $N_{i}$ represents the number of tokens with diverse granularities, and $W_{i}$ is the corresponding embedding dimension. Each $N_{i}$ contains a granular representation of its stage, produced by the federated block and forwarded to the next stage. Different values of the window $N_{o}$ are employed for each stage to model the interaction between each granularity and its neighbors. Specifically, in stages 1–3, the window values are 50, 400 and 2000 ms, respectively, ensuring the inclusion of all tokens within these intervals.

*(3) Jointing block*

The speech signal evolves progressively from stages 1 to 3, emphasizing distinct levels of features. To efficiently generate relevant features, we propose a jointing block. This mechanism is applied between every two stages, employing average pooling on the output values of each stage and determining their combined scale $Q_{i},i\in \left[1,2\right]$ ($Q_{1\;}$ of 50 ms and $Q_{2\;}$ of 250 ms) based on the granularity specific to each stage. Subsequently, linear projection and layer normalization are conducted to obtain the granular feature $s_{i},\;i\in \left[1,2\right]$ for $Stage_{i+1}$. This step ensures the aggregation of information from different minimum durations into tokens in $s_{i}$, with each token representing the granular feature of $Stage_{i+1}$. In the end, the merge scale $Q_{3\;}$ for the union block, applied to the output value $s_{3}$ of stage 3, is set to 1000 ms, approximating the number of words in the utterance sample. Word tags, representing coarse-grained features within words, are exempt from aggregation. This operation is expressed as

(15) $$s_{i}=Norm\left(AvgPool\left(s_{i},Q_{i}\right)O_{i}+m_{i}\right),$$

(16) $$c_{i}=Norm\left(c_{i}O_{i}+m_{i}\right),$$

where $AvgPool\left(s,Q\right)$ represents an average pooling layer performed on s with a window size and stride equal to Q; $O_{i}\in R^{W_{i}\times W_{i+1}}$ and $m\in R^{W_{i+1}}$ are parameters to be learned; $s_{i}$ and $c_{i}$ denote the outputs of the i-th stage; $x_{i+1}$ and $z_{i+1}$ denote the inputs of the next stage, $i\in \left\{1,2,3\right\}$.

The outputs of the last jointing block into the utterance stage are a stack of standard Transformer (ST) encoders to model the speech signal globally. The final output from the utterance stage will be aggregated along the temporal dimension and subsequently fed into the classifier. The classifier comprises two linear projections and an activation function in between to generate the classification result.

### 3.3. Loss Function

**Crucial cue stream loss:** We chose the categorical mean square error (MSE) loss as the objective function in the mask pathway. The MSE loss can be represented as

(17) $$Loss_{1}=\frac{1}{\alpha T}\overset{\alpha T}{\underset{i=1}{\sum }}{\left(y_{mask}-mask_{GT}\right)}^{2},$$

where $y_{mask}$ is the recovered information of the model’s output, and $mask_{GT}$ is the ground-truth of the corresponding masked part. α denotes the mask rate.

**Relationship stream loss:** We chose the categorical cross-entropy (CCE) loss as the objective function in the emotion pathway. The CCE loss can be represented as

(18) $$Loss_{2}=-\overset{C}{\underset{C=1}{\sum }}target_{emo}\cdot \log\left(y_{emo,C}\right),\;$$

where C denotes the number of emotion categories. $target_{emo}$ is the value of class C in the ground-truth label, and $y_{emo,C}$ is the probability of class C of the model output.

**Total loss:** For accommodating the training objectives of the speech emotion pathway and the mask pathway, the losses of the two tasks are merged with appropriate weighting. Throughout the training process, the model simultaneously learns two tasks related to SER and mask recovery. Therefore, the total loss function can be expressed as

(19) $$Loss_{total}=\left(1-\lambda \right)Loss_{1}+\lambda Loss_{2},\;$$

where λ is a hyperparameter that balances crucial cue stream loss and relationship stream loss.

## 4. Experimental Results

In this section, the experimental settings and implementation details are introduced. Then, to validate the effectiveness of the MelTrans model, we verify the performance of MelTrans in various aspects by conducting model-comparison experiments.

### 4.1. General Setting

**(1) Datasets:** Two datasets (IEMOCAP and EmoDB) are utilized for training and testing our MelTrans model.

**IEMOCAP** [27]: This dataset is recorded by 10 professional actors, consisting of 5 male and 5 female participants, and encompasses a total of 12 h of dialogue. It includes 10,039 sentences, which are annotated with four emotional labels: happy, neutral, angry and sad. The actors, each with diverse backgrounds, were trained to portray a wide range of emotional expressions, contributing to the dataset’s versatility and applicability across various speech emotion recognition tasks. Although some sentiments in the recordings may not be highly distinctive, which poses challenges in classifying emotional categories, the IEMOCAP dataset remains a powerful and universally applicable resource. Its realistic representation of emotional speech and balanced representation of gender make it a robust tool for advancing research in emotion recognition systems.

**EmoDB** [28]: This is one of the most popular databases in SER assignments. This dataset is a collection of 535 sentences, in which 302 sentences are of female emotion and 233 sentences are of male emotion.

**(2) Compared methods:** Numerous methods can be used for SER. Several representative methods and different versions of our proposed method are introduced as follows for the comparison experiments. All of the methods mentioned below have been fully implemented in the experimental phase.

**MLAnet** [41]: This network contains a multiscale low-level feature extractor and a multiunit attention module. The feature extractor minimizes the task-irrelevant information, which harms the performance of SER by applying an attention mechanism.

**ICAnet [15]**: This work proposes a novel framework integrating a cascaded attention network and an adversarial joint loss strategy for SER. The aim is to discriminate the confusing sentences by emphasizing more the emotions that are difficult to be correctly classified.

**TRIN** [31]: TRIN fully considers the underlying hierarchy of a phonetic structure and its associations between various modalities under sequential temporal guidance. This model assumes that all modalities are related; it infers the dependency relationship between the semantic information from the temporal level in a flexible order.

**SCAR-NET** [42]: In this paper, the authors propose SCAR-NET, an improved CNN, to extract emotional features from speech signals for SER. The model extracts spectral, temporal and spectral–temporal correlation features through three parallel paths. Then, split–convolve–aggregate residual blocks are designed for multibranch deep feature-learning.

**(3) Evaluation metrics:** The weighted accuracy (WA) and the unweighted accuracy (UA) are used for the evaluation. Given that WA and UA may not reach their maximum values in the same model, the average of WA and UA is calculated as the final evaluation criterion.

The confusion matrix is a square table that is used to define the performance of a classification algorithm. The performance is summarized and visualized through the confusion matrix. The numbers on the diagonal of the confusion matrix represent the right predictions; the numbers not on the diagonal represent wrong predictions. A high percentage of predictions on the diagonal suggests the high accuracy of an algorithm. The confusion matrix reflects to what degree the algorithm makes confused predictions.

### 4.2. Implementation Details

The proposed model is implemented in the following manner. The number of training epochs is set to 200 and 500 for IEMOCAP and EmoDB, respectively. The balance hyperparameter λ is set as 0.7, the mask rate to 20%, the learning rate to 0.001 and the batch size to 16. All the experiments are performed on one Nvidia TITAN GPU using the PyTorch toolbox.

### 4.3. Experimental Results and Analysis

We compare our method with several of the best-performing methods on the individual datasets and analyze their performance. It is important to note that these comparisons involve evaluating methods on two different datasets, each utilizing varying comparison methodologies. These variations may influence the direct comparability of the results. Therefore, the performance differences should be interpreted with caution, acknowledging the potential impact of these varying conditions on the outcomes.

*(1) Performance comparison on the IEMOCAP dataset*

We first conduct experiments using several current SER methods on the IEMOCAP dataset. The results are listed in Table 1. TRIN achieves excellent performance by considering the underlying hierarchy of the phonetic structure and its associations between various modalities under sequential temporal guidance. ICAnet realizes impressive performance by integrating a cascaded attention network and an adversarial joint loss strategy. Based on the Transformer architecture, MelTrans shows improvement compared to the state-of-the-art methods, which indicates the capacity of our model. Compared with previous methods, our model reaches a considerable accuracy of 76.52%, suggesting that our model is successful in exploiting the invariant cues and long-dependent semantic relationships in voice signals.

*(2) Performance comparison on the EmoDB dataset*

The proposed method is also compared with [2,45,46], AMSNet, ICAnet and SCAR-NET on the EmoDB dataset. Table 2 displays the results and comparisons. Our method exhibits 92.5% accuracy. SCAR-NET achieves excellent performance by utilizing split–convolve–aggregate residual blocks for multibranch deep feature-learning, implying the significance of multiscale information for SER to achieve improved results. Our method exploits the information over long distances from speech signals, which is proven to yield improved performance. Compared with LSTM- or CNN-based models, Transformer-based models achieve expressive performance mainly because the Transformer architecture intrinsically possesses the capacity to exploit the long-range-dependent semantic relationships hidden among all tokens. In general, MelTrans yields excellent performance among the compared methods.

### 4.4. Analysis of the Dual-Stream Design and Discussion

**Figure 3:**
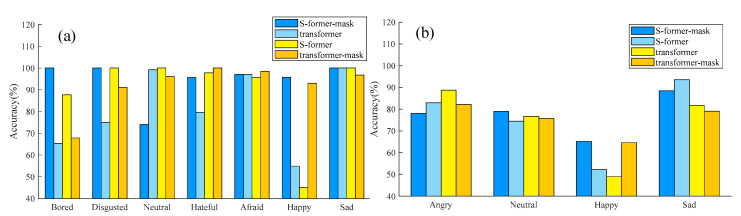
Detailed performance on different emotions among four variants. (**a**) Results on the EmoDB dataset. (**b**) Results on the IEMOCAP dataset.

In this section, we undertake a thorough study to analyze the segments of our MelTrans model and present our findings. Given that the relationship stream (S-former) is derived from the standard Transformer (ST) architecture, we opt to conduct a comparative analysis between them. That is, only a separate ST model is used in the SER task. In conducting this study, we aim to provide insights into the individual contributions of different segments (dual-stream design) within our model, thereby facilitating a deep understanding of its functioning and effectiveness in emotion recognition tasks. Such analyses contribute to the ongoing refinement and optimization of state-of-the-art models in the field of SER. Specifically, our study focuses on the ablation of the crucial cue stream and the relationship stream. The results, as depicted in Figure 3, illustrate that MelTrans demonstrates robust performance across the IEMOCAP and EmoDB datasets. Notably, our analysis highlights the significance of the crucial cue stream in enhancing the overall performance of the model. This finding reveals the critical role played by the crucial cue stream in the MelTrans architecture.

**Detailed analysis of each class:** Figure 3 illustrates the accuracy of different variants evaluated on the EmoDB and IEMOCAP datasets. In Figure 3a, the results on the EmoDB dataset indicate that MelTrans demonstrates a commendable performance across most emotion categories, with the exception of neutral emotion classification, where its performance appears not so good. For the S-former model depicted in Figure 3a, the challenge lies in discriminating between happy and neutral speech emotions. From the results on the IEMOCAP dataset shown in Figure 3b, the recognition accuracy for happy emotions tends to be relatively lower than those for other emotion categories. The S-former model and the MelTrans model consistently outperform the ST model across various emotions on both datasets. The effectiveness of the word-tokens branch in the MelTrans model is particularly evident in addressing the challenge of emotional subtleties. By converting speech signals into word tokens, this branch captures and identifies subtle variations in vocal attributes such as tone, pitch and intensity. These minute but crucial features are often overlooked, yet they play a decisive role in distinguishing fine-grained emotional differences. As mentioned in our key insights, the subtle discrepancies between emotions often reside in inconspicuous but significant long-range dependencies. By uncovering these fine-grained, determinative features, the word-token branch enhances the model’s sensitivity to emotional nuances, particularly in recognizing emotions like ‘happy’ and ‘sad’, where minor tonal variations are key indicators of emotional states. The results presented in Figure 3 demonstrate that this branch significantly contributes to the model’s overall performance, particularly by extracting determinative long-range dependencies that improve the accuracy of emotion recognition for these challenging and subtle emotions.

**Figure 4:**
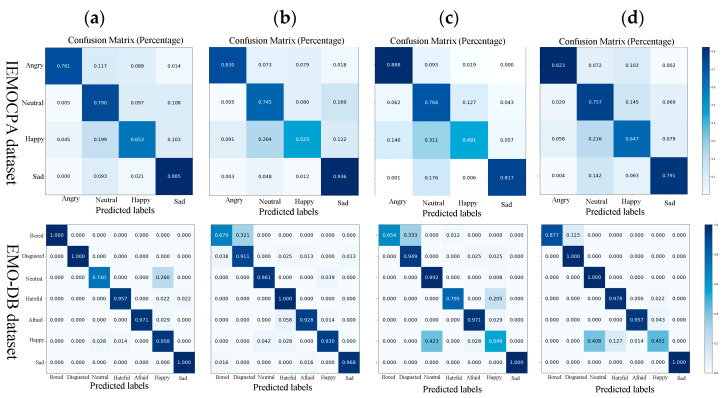
Confusion matrix analysis of model variants. First row: IEMOCAP dataset. Second row: EmoDB dataset. (**a**) Relationship stream. (**b**) ST+crucial cue stream. (**c**) ST. (**d**) MelTrans.

**Confusion matrix analysis:** The confusion matrix shown in Figure 4 illustrates that the S-former model struggles with distinguishing between neutral and happy emotions, leading to potential misclassifications that could result in inappropriate responses or misunderstandings in intelligent voice assistants, thereby affecting their effectiveness in real-world applications. On the IEMOCAP dataset, the S-former model and the MelTrans model exhibit superior performance compared with the ST model, particularly across different emotional categories. Further analysis in Figure 4 reveals a mutual interference between neutral and happy emotions, indicating that misclassifications between these two emotions are recurrent and tend to hinder accurate emotion recognition. Therefore, the ambiguous boundary between happy and neutral speech emotions often poses a significant challenge, resulting in reduced accuracy in SER. These observations underline the complexity inherent in recognizing subtle emotional nuances.

**Figure 5:**
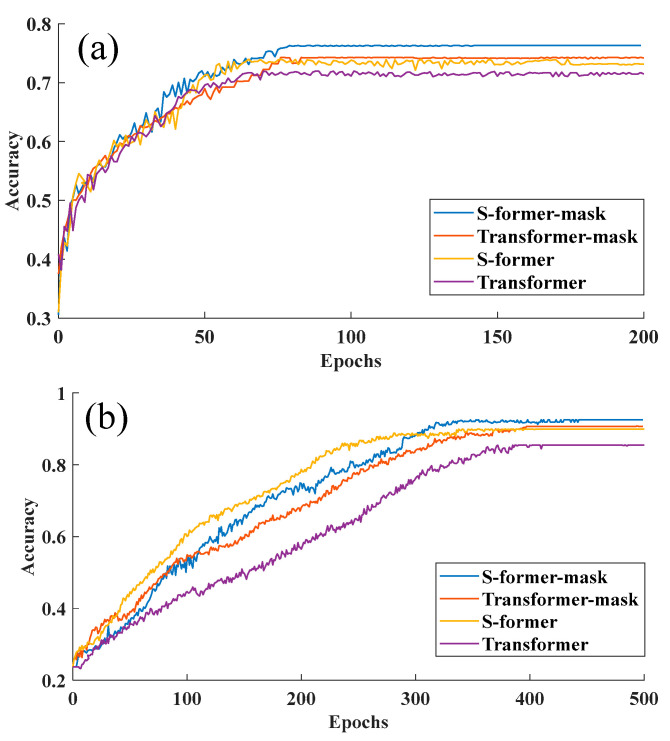
Training curves of four model variants on two datasets. (**a**) On the EmoDB dataset. (**b**) On the IEMOCAP dataset.

**Effectiveness of the dual-stream design:** To verify the effectiveness of the dual-stream design, we opt for a multitask model configuration, comprising a nonmask model in conjunction with a masked component, to conduct a comparative analysis. This design delineates the nonmask model to exclusively handle a SER task, with the absence of a mask model to provide auxiliary analysis on specific key features. Figure 5 shows that the performance of the MelTrans model with the masking strategy is consistently outstanding compared to the others. Furthermore, the multitask performance of the S-former submodel and the mask-word model emerges as optimal. The mask-sequence branch of the MelTrans model plays a pivotal role by simulating noisy environments through masking certain speech fragments. This allows the model to focus on essential features and maintain clarity even with background interference. As shown in Figure 5, this approach reduces misclassifications between similar emotions like ‘neutral’ and ‘happy’ under noisy conditions, significantly enhancing the model’s ability to accurately identify the speaker’s emotional state in challenging acoustic environments.

On the IEMOCAP dataset, as depicted in Figure 5, the multitask model incorporating a mask demonstrates superior performance compared to the standalone transform and S-former models. The results in Table 3 reveal that the multitask model exhibits an approximately 3% performance enhancement over its single-task counterpart. This finding underscores the robust capability of the crucial cue stream in capturing crucial features, thereby significantly aiding the overall effectiveness of emotion recognition. For the IEMOCAP dataset, the outcomes presented in Table 4 indicate the commendable performance of our MelTrans model in recognizing the sad speech emotion. On the EmoDB dataset, the accuracy of MelTrans and the S-former model is evaluated for each speech emotion. The results are shown in Table 5, from which the accuracy for emotions such as bored, disgusted and sad is satisfactory, whereas the accuracy for the neutral and happy emotions is comparatively lower. The relatively diminished scores for the happy emotion can be attributed to MelTrans’s enhanced generalization across other emotions, coupled with a potential imbalance in the representation of the angry and neutral emotions. The model accuracy curve during training is analyzed in Figure 5. The ST model performs slightly worse on the EmoDB and IEMOCAP datasets. Consequently, the MelTrans model may exhibit a propensity to acquire less-discriminative information of these specific emotion categories, resulting in lower accuracy for them. In conclusion, the employment of the relationship stream emerges as a pivotal factor contributing to a notable enhancement for SER. Insights gleaned from Figure 5 and Table 5 on the EmoDB dataset indicate the commendable efficacy of the crucial cue stream.

## 5. Limitations and Future Work

The current work has some limitations that offer opportunities for future research. Firstly, our experiments were conducted on two indoor German datasets, IEMOCAP and EmoDB. While these datasets are widely recognized and provide valuable benchmarks, they inherently limit the generalizability of our findings to other languages, acoustic environments and spontaneous speech scenarios. Future work should aim to validate the proposed method on more diverse datasets, including those with different languages, cultures and environmental conditions, to ensure broader applicability. Secondly, the use of a Transformer-based model, although effective in capturing long-range dependencies and critical cues in speech data, comes with significant computational complexity. This may limit its efficiency, particularly in real-time applications. Future research will focus on optimizing the model’s architecture to reduce computational load, possibly through model pruning, quantization, or the development of lightweight versions of the Transformer model. These efforts will enhance the practical deployment of the model in real-time emotion recognition systems. Finally, while our approach demonstrates a strong performance on the datasets used, there is potential for further improvement in the feature extraction process. Integrating additional modalities, such as facial expressions or physiological signals, could enrich the model’s understanding of human emotions and lead to more robust predictions. Expanding the method to multi-modal emotion recognition systems is another promising direction for future work.

## 6. Conclusions

In this work, we propose the significant characteristics for SER. We reveal two findings in voice signals: crucial cues of emotions and minuscule discrepancies in different emotions. We consider that making full use of the two findings is of great significance to facilitate SER. Thus, we propose an efficient method dubbed MelTrans with a dual-stream design. The crucial cue stream extracts the crucial cues of speech via a masking strategy. The relationship stream aggregates the multiscale information of voice signals. The Transformer architecture is chosen as the backbone to reveal the long-range-dependent semantic relationships in speech signals. We evaluate our MelTrans on two SER datasets. The experimental results demonstrate that our approach can recognize crucial cues and long-range-dependent relationships in speech signals. In the future, we will focus on building a lightweight network for the speech emotional recognition tasks.

## Figures and Tables

**Table 1:** Comparison results on the IEMOCAP dataset in terms of WA and UA.

Methods	Backbone	WA (%)	UA (%)	Acc (%)
FENT [43]	CNN	71.84	73.88	72.86
MLT-DNet [37]	CNN	73.22	72.88	73.00
Zheng et al. [33]	ResNet	71.64	72.70	72.17
AMSNet	ResNet	69.22	70.51	69.87
ISNet	ResNet	70.43	65.02	67.73
SpeechFormer++	Transformer	70.50	71.5	71.00
SDT [44]	Transformer	73.82	74.08	73.95
ICAnet	Transformer	**82.68**	**82.67**	**82.68**
MelTrans (Ours)	Transformer	76.50	76.54	76.52

**Table 2:** Comparison results on the EmoDB dataset in terms of WA, UA.

Methods	Backbone	WA (%)	UA (%)	Acc (%)
MLT-DNet	CNN	90.90	89.10	90.00
Jiang et al. [45]	CNN	87.9	86.7	87.30
ICAnet	CAN	91.58	88.76	90.17
AMSNet	ResNet	88.34	88.56	88.45
SCAR-NET	Transformer	-	-	**96.45**
DeepESN [46]	Transformer	87.89	87.14	87.51
HuBERT [2]	Transformer	-	-	89.00
MelTrans (Ours)	Transformer	92.47	92.50	92.52

**Table 3:** Model variants on the EmoDB and IEMOCAP datasets.

Dataset	Model Variants	SpeechFormer	Mask	Recall	F1	Accuracy
EmoDB dataset	ST stream	**×**	**×**	0.864	0.844	0.854
	S-former stream	**√**	**×**	0.900	0.899	0.899
	ST+crucial cue stream	**×**	**√**	0.910	0.905	0.906
	MelTrans	**√**	**√**	**0.927**	**0.926**	**0.925**
IEMOCAP dataset	ST stream	**×**	**×**	0.741	0.729	0.717
	S-former stream	**√**	**×**	0.758	0.740	0.732
	Transformer-mask	**×**	**√**	0.754	0.755	0.743
	MelTrans	**√**	**√**	**0.776**	**0.775**	**0.766**

**Table 4:** Performance on different speech emotions on the IEMOCAP dataset.

Method	Angry (%)	Neutral (%)	Happy (%)	Sad (%)
S-former	82.95	74.55	52.28	93.60
MelTrans	78.15	79.00	65.32	88.54

**Table 5:** Performance on different emotions on the EmoDB dataset.

Method	Bored (%)	Disgusted (%)	Neutral (%)	Hateful (%)	Afraid (%)	Happy (%)	Sad (%)
S-former	100.00	100.00	74.02	95.65	97.10	95.77	100.00
MelTrans	100.00	100.00	100.00	97.78	95.65	45.40	100.00

## Data Availability

The data presented in this study are available in IEMOCAP and EmoDB datasets, reference number 27 and 28. These data were derived from the following resources available in the public domain IEMOCAP (https://sail.usc.edu/iemocap/).
